# Fatigue, Social Support, and Depression in Individuals With Coronary Artery Disease

**DOI:** 10.3389/fpsyg.2021.732795

**Published:** 2021-10-20

**Authors:** Nijole Kazukauskiene, Adomas Bunevicius, Julija Gecaite-Stonciene, Julius Burkauskas

**Affiliations:** Laboratory of Behavioral Medicine, Neuroscience Institute, Lithuanian University of Health Sciences, Palanga, Lithuania

**Keywords:** fatigue, social support, depression, coronary artery disease, acute coronary sindrome, cardiac rehabilitation

## Abstract

**Background:** Given that approximately one-third of individuals with coronary artery disease (CAD) remain severely fatigued after completion the cardiac rehabilitation, it is necessary to identify reliable intervention targets aimed at reducing fatigue. Perceived social support is closely linked to health outcomes and depressive symptoms in individuals with CAD. However, to our knowledge, the relationship between subjective fatigue levels and social support in those with CAD has not been analyzed.

**Objective:** We aimed to examine the associations between perceived social support and subjective fatigue levels in individuals with CAD with and without depression symptoms.

**Methods:** This cross-sectional study was comprised of 1,036 participants with CAD (57±9years, 77% men) 1–2weeks after acute coronary syndrome (ACS). Participants completed the Hospital Anxiety and Depression scale (HADS), Multidimensional Fatigue Inventory-20 (MFI-20), and the Multidimensional Scale of Perceived Social Support (MSPSS).

**Results:** In total, 12% (*n*=129) of study participants had elevated depression symptoms (HADS score≥8). In individuals with CAD and depressive symptoms, after adjustment for sex, age, New York Heart Association (NYHA) functional class, and anxiety, linear regression analyses showed significant inverse associations between higher social support from others and general, physical fatigue as well as reduced activity and motivation (*p*<0.001). Following the same method of statistical analysis and control in non-depressed individuals with CAD (88%), social support from family was inversely linked to mental fatigue (*p*’s<0.05). Similarly, social support from friends was significantly associated with lower general, physical, and mental fatigue as well as reduced activity, while social support from others was significantly associated with lower general and mental fatigue (*p*’s<0.001). The overall higher total support was linked with reduced motivation (*p*<0.05) in the depressed study participants, while there was lower general and mental fatigue (*p*<0.05) in non-depressed individuals.

**Conclusion:** The results of this study suggest that fatigue and its features could be associated by the perceived social support in individuals with CAD following ACSs. While in individuals with CAD and depressive symptoms, greater subjective fatigue is associated with less perceived social support from others, higher levels of subjective fatigue in non-depressed individuals with CAD are significantly associated with reduced perceived social support from friends.

## Introduction

Coronary artery disease (CAD) is one of the most common cardiovascular illness affecting people all over the world and is the leading cause of death in both developed and developing countries (Malakar et al., 2019). Extensive research has focused on identifying prognostic factors of individuals with CAD who remain at high risk for recurrent cardiac events (Miller and Shaw, 2006; Batacan Jr. et al., 2015). It is well established that perceived social support is closely linked to health outcomes in individuals with CAD. Specifically, lack of social support is associated with increased morbidity and mortality in individuals with CAD (Mookadam and Arthur, 2004; Barth et al., 2010; Compare et al., 2013) and with worse health-related quality of life (Staniute et al., 2013).

Fatigue is a common symptom to various medical and mental conditions, including cancer, multiple sclerosis, major depression, schizophrenia, and heart related conditions, which may impair quality of life (Chung et al., 2014; Hedlund et al., 2015; Mohandas et al., 2017; Rottoli et al., 2017). Considering the context, CAD is not an exception, since fatigue is a common disturbing symptom in individuals with this particular diagnosis, linked to poor cognitive performance (Burkauskas et al., 2017) and worse health related quality of life (Staniute et al., 2014). In fact, fatigue is often present as a core somatic symptom of depression that is one of the most common comorbid mental health conditions in those with CAD (Smith et al., 2008; Alsen and Brink, 2013). Previous research in 1,470 individuals with CAD has shown a strong and positive relationship between depressive symptoms and reported fatigue (Bunevicius et al., 2011b). Given that approximately one-third of individuals with CAD who have completed the cardiac rehabilitation program remain severely fatigued, it is necessary to identify early and reliably the prevalence of fatigue, its features and related co-occurring problems in those individuals, in order to adjust the cardiac rehabilitation program accordingly.

For individuals with fatiguing conditions like CAD, effective care involves measuring the severity, associated factors, incidence, duration, nature, and form of fatigue, as this may help to individualize and track the success of rehabilitation based on minimizing fatigue symptoms (van Geffen et al., 2015). Even though fatigue and perceived social support are closely linked to health outcomes and depression in this clinical population (Smith et al., 2008; Barth et al., 2010; Bunevicius et al., 2011a), it remains unclear whether perceived social support is associated with fatigue in the context of depression symptoms.

So far, an interplay between perceived social support and subjective fatigue levels received little attention, and, to our knowledge, has not been analyzed in scientific medical literature. Thus, the aim of this study was to examine the associations between perceived social support and subjective fatigue in a large sample of individuals with established CAD with and without depressive symptoms.

## Materials and Methods

### Study Procedure

During the period from 2014 to 2019, a total of 1,143 participants with CAD were invited to participate in this study. All the participants were admitted to the rehabilitation program within 1–2weeks following the treatment for acute coronary syndrome (ACS; i.e., myocardial infarction or angina pectoris). Our inclusion criteria were: (a) confirmed CAD diagnosis and recent ACS as identified by a study cardiologist; (b) no history of arrhythmic disorder and/or implantation of a cardioverter defibrillator; (c) comprehension of Lithuanian language; and (d) age 18–80years. Our exclusion criteria were: (a) unstable cardiovascular condition (*n*=52), (b) severe comorbidities (e.g., kidney failure or musculoskeletal disease; *n*=28), and (c) unwillingness to participate in the study (*n*=27). Thus, the final sample was comprised of 1,036 participants with CAD (57±9years, 77% men). All participants were subjected to standard evaluation and treatment for the secondary prevention of CAD according to the existing guidelines (Gibbons et al., 2002; Piepoli et al., 2010; Fletcher et al., 2013; O'Gara et al., 2013).

Within 2days of admission to the rehabilitation program and after providing written consent, all study participants were evaluated for demographic information (i.e., age, sex, education, and marital status) and clinical factors [i.e., New York Heart Association (NYHA) functional class, history of ACS, and angina pectoris class]. Participants also completed questionnaires assessing their subjective fatigue levels (the Multidimensional Fatigue Inventory-20, MFI-20; Smets et al., 1995), and mental distress symptoms were measured using the Hospital Anxiety and Depression scale (HADS; Zigmond and Snaith, 1983). Study participants also completed a questionnaire assessing perceived social support (Multidimensional Scale of Perceived Social Support, MSPSS; Zimet et al., 1990).

All procedures conducted in a current research involving human subjects were in compliance with the ethical principles of the Biomedical Research Ethics Committee for Biomedical Research at Lithuanian University of Health Sciences and conformed to the principles outlined in the Declaration of Helsinki. Informed consent was attained from each participant agreeing to be enrolled in the study.

### Measures

#### Multidimensional Fatigue Inventory-20

Fatigue severity was measured using the MFI-20 (Smets et al., 1995). The MFI is a 20-item scale designed to evaluate (1) general fatigue, (2) physical fatigue, (3) mental fatigue, (4) reduced activity, and (5) reduced motivation. Each domain consists of four items with possible answers on a five-point (1=“yes, that is true”; 5=“no, that is not true”) Likert type scale. General fatigue domain is composed of general statements about fatigue and reduced functioning, covering physical and psychological aspects of fatigue. Physical fatigue concerns physical feelings related to fatigue. Mental fatigue relates to cognitive functioning, such as concentration difficulties. Reduced activity subscale evaluates the impact of psychological and physical factors on the activity level. The lack of motivation to start an activity is reflected in the reduced motivation subscale. Individual subscale scores (range 4–20) are calculated as the sum of item ratings, while a total fatigue score (range 20–100) is calculated as the sum of scores of all the five subscales. Higher scores indicate a higher level of fatigue. The psychometric characteristics of Lithuanian version of MFI were documented in individuals with CAD (Gecaite-Stonciene et al., 2020). The internal consistency values were reasonable for the MFI-20 total fatigue score in four of the five domains: general fatigue, physical fatigue, reduced activity, reduced motivation, and mental fatigue Cronbach’s range: 0.55–0.912 (Taber, 2018).

#### Multidimensional Scale of Perceived Social Support

Perceived social support was assessed using the MSPSS (Zimet et al., 1990). The MSPSS assesses the perceived adequacy of social support from family, friends, and significant others as rated by the participants. The MSPSS is comprised of 12 items assessing social support scored on a seven-point Likert scale ranging from “very strongly disagree” (1) to “very strongly agree” (7). For the total score, ratings are first added and then divided by 12 to retrieve the averaged score. Total scores range from 1 to 7, with higher scores indicating higher levels of perceived available social support (Zimet et al., 1990). The psychometric properties of the scale are adequate, with Cronbach α for total scale ranging from 0.81 to 0.94 in previous studies (Pedersen et al., 2009; Staniute et al., 2013). Cronbach’s alpha coefficients for the present sample were 0.75 for the family subscale, 0.89 for the friends subscale, 0.80 for the others subscale, and 0.91 for the full scale.

#### Hospital Anxiety and Depression Scale

The HADS is a self-report questionnaire consisting of 14 items that are used to evaluate anxiety (HADS-A) and depression (HADS-D) symptoms (Zimet et al., 1990). Total scores on the HADS-A and HADS-D ranged from 0 to 21, with higher scores indicating higher anxiety or depressive symptoms. The Lithuanian version of HADS was documented to have good psychometric characteristics in individuals with CAD (Bunevicius et al., 2011a). Acceptable internal consistency of HADS was observed in our study sample (HADS-D Cronbach’s α=0.73; HADS-A Cronbach’s α=0.83).

### Statistical Analysis

Statistical analysis was performed using the Statistical Package for Social Sciences (SPSS) 17.0 for Windows (Chicago, Illinois). Data are expressed as mean±SD for variables with Gaussian distribution and as number (percent) for qualitative variables. We first calculated the means and frequencies for socio-demographic, clinical factors, mental distress, fatigue, and perceived social support scores. Then, a *t*-test for independent samples and *χ*^2^ criteria for categorical variables were used for the comparisons between means and frequencies.

Later, regression analyses were performed for those with and without depression symptoms separately. We employed univariate regression analysis to determine unadjusted associations of perceived social support scores with scores on subjective fatigue. All variables that were statistically significantly (*p*<0.05) associated with fatigue domain in univariate analyses were subsequently included in respective multivariable regression models. We used multivariable linear regression analyses to determine if perceived social support remained associated with subjective fatigue after adjustment for sex, age, NYHA functional class, and anxiety symptoms that significantly correlated in univariate models.

## Results

Socio-demographic, clinical data, mental distress, perceived social support, and subjective fatigue characteristics of all 1,036 participants and results stratified by depression symptoms are presented in Table 1. In short, the average age of participants was 57.3±9.2years. The majority of the participants (77%, *n*=798) were men. In total, 8% (*N*=81) of the participants had I NYHA functional class, 79% (*N*=817) of the participants had II NYHA functional class, and the remaining 13% (*N*=138) of the participants were classified to the III NYHA functional class. No subjects were classified as having IV NYHA functional class. Moderate to severe anxiety symptoms (HADS-A score≥8) were found in 32% (*N*=335) of participants. Further, approximately 88% (*n*=907) of individuals did not show significant depressive symptoms, while the remaining 12% (*n*=129) were classified as having significant depressive symptoms. Individuals with significantly expressed depressive symptoms were more likely to be older (61±9 vs. 57±9), female (40 vs. 21%), had NYHA III functional class (20 vs. 12%), and had significantly higher anxiety symptoms (HADS-A≥8, 66 vs. 28%), lower perceived social support (5.4±1.2 vs. 6.1±1.0), and higher levels of subjective fatigue (13.8±3.2 vs. 10.3±3.9). Since major significant differences emerged in terms of anxiety, social support, and fatigue in those with and without markedly expressed depression symptoms, separate regression analyses were performed to emphasize possible group differences.

**Table 1:** Characteristics of all individuals after acute coronary syndromes (ACSs) at inclusion stratified by depression symptoms.

Characteristics	All individuals (*n*=1,036)	Markedly expressed symptoms of depression	No or mild symptoms of depression	*p* value
		HADS-D≥8 (*n*=129)	HADS-D<8 (*n*=907)	
Age, years (mean±SD)	57.3±9.2	60.8±9.0	56.8±9.1	**<0.001**
Sex, *n* (%)				**<0.001**
Men Women	798 (77.0) 238 (23.0)	78 (60.5) 51 (39.5)	720 (79.4) 187 (20.6)	
Marital status, *n* (%)				0.245
Married Separated Widowed Single Cohabitation	837 (80.8) 75 (7.2) 75 (7.2) 19 (1.8) 30 (2.9)	96 (74.4) 14 (10.9) 13 (10.1) 2 (1.6) 4 (3.1)	741 (81.7) 61 (6.7) 62 (6.8) 17 (1.9) 26 (2.9)	
Education, *n* (%)				0.095
Up to 8years High school graduate College/University degree	86 (8.3) 506 (48.8) 444 (42.9)	17 (13.2) 58 (45.0) 54 (41.9)	69 (7.6) 448 (49.4) 390 (43.0)	
Living alone, n (%)	102 (9.8)	16 (12.4)	86 (9.5)	0.297
Diagnosis, *n* (%)				0.124
Angina pectoris Acute myocardial infarction Previous MI	280 (27.0) 654 (63.1) 102 (9.8)	42 (27.0) 71 (55.0) 16 (12.4)	238 (26.2) 583 (64.3) 86 (9.5)	
NYHA class, *n* (%)				**0.003**
I II III	81 (7.8) 817 (78.9) 138 (13.3)	3 (2.3) 100 (77.5) 26 (20.2)	78 (8.6) 717 (79.1) 112 (12.3)	
Mental distress				**<0.001**
HADS-A≥8, n (%)	335 (32.3)	85 (65.9)	250 (27.6)	
Perceived social support scores, mean score±SD				
Social support from family	6.4±1.1	5.9±1.4	6.4±1.0	**<0.001**
Social support from friends	5.6±1.4	4.7±1.7	5.7±1.4	**<0.001**
Social support from others	6.1±1.2	5.5±1.5	6.2±1.2	**<0.001**
Total social support	6.0±1.1	5.4±1.2	6.1±1.0	**<0.001**
MFI-20 scores, mean score±SD				
Global fatigue	10.8±4.0	13.8±3.2	10.3±3.9	**<0.001**
Physical fatigue	11.7±4.3	15.0±3.3	11.2±4.2	**<0.001**
Activity reduction	12.3±4.0	15.0±3.1	11.9±3.9	**<0.001**
Motivation reduction	9.9±3.4	12.3±3.0	9.5±3.3b	**<0.001**
Mental fatigue	9.8±4.0	12.9±3.7	9.3±3.9	**<0.001**

MFI-20, Multidimensional Fatigue Inventory-20; NYHA, New York Heart Association; HADS, Hospital Anxiety and Depression Scale; HADS-A, HADS Anxiety subscale; HADS-D, HADS depression subscale, *p* value of probability for comparison between groups (bolded numbers indicate significant differences, *p*<0.05); and data presented as *n* (%) and as mean±SD, significant values in bold.

Table 2 presents the association between the subscales of the MFI-20 and MSPSS stratified by depressive symptoms. In depressed individuals with CAD, after adjustment for sex, age, NYHA functional class, and anxiety, linear regression analyses showed significant association between social support from others and general fatigue (*β*=−0.177, *p*=0.043), physical fatigue (*β*=−0.211, *p*=0.016), reduced activity (*β*=−0.261, *p*=0.004), and reduced motivation (*β*=−0.183, *p*=0.041). Following the same method of statistical analysis and control in individuals with CAD and without markedly expressed depression symptoms, we found significant associations between higher social support from family and mental fatigue (*β*=−0.073, *p*=0.014). Similarly, perceived social support from friends was significantly linked with lower general fatigue (*β*=−0.107, *p*<0.000), physical fatigue (*β*=−0.096, *p*=0.002), reduced activity (*β*=−0.084, *p*=0.011), and mental fatigue (*β*=−0.105, *p*<0.000); while perceived social support from others was significantly associated with general fatigue (*β*=−0.071, *p*=0.018) and mental fatigue (*β*=−0.080, *p*=0.007). The overall higher total support was linked with lower general (*β*=−0.095, *p*=0.002) and mental (*β*=−0.104, *p*<0.000) fatigue in non-depressed individuals and with reduced motivation (*β*=−0.187, *p*=0.035) in study participant with markedly expressed symptoms of depression.

**Table 2:** Association between the Perceived social support and the subscales of the MFI-20 stratified by depression symptoms.

Group	MFI-20	Models	*R* ^2^(<0.001)	Social support from family	Social support from friends	Social support from others	Total social support
With depression symptoms	General fatigue	U		**−0.199 (0.024)**	−0.073 (0.414)	**−0.187 (0.034)**	**−0.184 (0.037)**
		M	0.078–0.107	−0.162 (0.069)	−0.046 (0.606)	**−0.177 (0.043)**	−0.152 (0.084)
	Physical fatigue	U		**−0.208 (0.018)**	−0.070 (0.432)	**−0.234 (0.008)**	**−0.205 (0.020)**
		M	0.077–0.117	−0.162 (0.068)	−0.058 (0.513)	**−0.211 (0.016)**	−0.171 (0.051)
	Reduced activity	U		−0.104 (0.242)	−0.020 (0.824)	**−0.233 (0.008)**	−0.143 (0.105)
		M	0.019–0.084	−0.121 (0.188)	−0.023 (0.804)	**−0.261 (0.004)**	−0.160 (0.076)
	Reduced motivation	U		−0.119 (0.181)	−0.130 (0.142)	**−0.190 (0.031)**	**−0.182 (0.039)**
		M	0.061–0.080	−0.127 (0.161)	−0.147 (0.102)	**−0.183 (0.041)**	**−0.187 (0.035)**
	Mental fatigue	U		−0.116 (0.189)	−0.015 (0.865)	−0.065 (0.465)	−0.078 (0.382)
		M	0.224–0.225	−0.045 (0.584)	0.017 (0.833)	−0.028 (0.729)	−0.020 (0.801)
Without depression symptoms	General fatigue	U		**−0.075 (0.024)**	**−0.138 (<0.001)**	**−0.108 (0.001)**	**−0.131 (<0.001)**
		M	0.190–0.198	−0.052 (0.082)	**−0.107 (<0.001)**	**−0.071 (0.018)**	**−0.095 (0.002)**
	Physical fatigue	U		−0.021 (0.522)	**−0.119 (<0.001)**	−0.057 (0.089)	**−0.084 (0.011)**
		M	0.131–0.140	−0.004 (0.895)	**−0.096 (0.002)**	−0.025(0.426)	−0.055 (0.076)
	Reduced activity	U		−0.018 (0.592)	**−0.096 (0.004)**	−0.047 (0.155)	**−0.068 (0.039)**
		M	0.073–0.079	−0.006 (0.859)	**−0.080 (0.013)**	−0.028 (0.387)	−0.050 (0.123)
	Reduced motivation	U		−0.009 (0.785)	**−0.084 (0.011)**	−0.040 (0.230)	−0.057 (0.085)
		M	0.145–0.148	0.008 (0.790)	−0.059 (0.057)	−0.005 (0.874)	−0.026 (0.400)
	Mental fatigue	U		**−0.099 (0.003)**	**−0.143 (<0.001)**	**−0.121 (<0.001)**	**−0.146 (<0.001)**
		M	0.215–0.220	**−0.073 (0.014)**	**−0.105 (<0.001)**	**−0.080 (0.007)**	**−0.104 (<0.001)**

U, univariate regression model; M, multivariable regression model [adjustment for sex, age, NYHA functional class, and anxiety (as measured by Hospital Anxiety and Depression Scale, Anxiety subscale)], significant values are presented in bold; and MFI-20, Multidimensional Fatigue Inventory-20.

## Discussion

Our results indicate that perceived social support is an important determinant of subjective fatigue levels in individuals with CAD following ACS. This was the first study to explore how this association is dependent on depression symptoms. Based on our findings, social support in both participants with and without depression was a factor directly associated with the subjective fatigue. Recent findings imply that, while in individuals with CAD with markedly expressed depression symptoms higher levels of subjective fatigue are associated with mostly social support from others, higher levels of subjective fatigue in non-depressed individuals with CAD are significantly associated with mostly reduced social support from friends. Furthermore, these associations were independent of various potential confounders, such as sex, age, NYHA functional class, and anxiety.

Since subjective fatigue and perceived social support are closely linked to health outcomes in individuals with CAD (Smith et al., 2008; Barth et al., 2010; Burkauskas et al., 2017), it is important to determine the presence of fatigue and its features, and how they could be predicted by the perceived social support in those individuals early and accurately, as well as adapt the rehabilitation program accordingly. Since one of the main symptoms of depression is fatigue (Bunevicius et al., 2012), a further intervention that focuses on the treatment of depression is likely to be beneficial. In terms of subjective fatigue level, the study by van Geffen et al. (2015) has reported the prevalence of severe fatigue remained higher in individuals with CAD after rehabilitation than in healthy individuals. This may suggest that the cardiac rehabilitation program might be inadequate for these individuals in terms of fatigue.

Given the cross-sectional structure of the research by Cho et al. (2019), it is possible that depressed, insomniac, or fatigued older adults become socially isolated and also poorly rate the quality of their social relationships. It is shown that higher levels of social support have been linked to decreased signs of loneliness and depression. A higher level of social support is significantly positively associated with positive coping styles and consequently predicts fewer depressive symptoms (Chen et al., 2019). Considering the current situation with COVID-19 and its possible long term outcomes on people’s mental health, studying social support and its related characteristics, may become of great importance, since loneliness in these difficult times has affected almost every age group (Berg-Weger and Morley, 2020; Morgul and Bener, 2020; Patel and Clark-Ginsberg, 2020; Xu et al., 2020; Zubatsky et al., 2020; Losada-Baltar et al., 2021).

Considering the mental health of senior citizens, the findings of the study by Tariq et al. (2020) reveal the difficulties faced by this age group. Particularly, the findings indicate that seniors wifvth physical disabilities are more likely to experience symptoms of depression in later years of life if they lack any of the three dimensions of perceived social support. This emphasizes the significance of the perceived support from family, friends, and significant others in preventing physical and cognitive health obstacles in later life. Depending on the findings, there could be an opportunity to reduce or alleviate fatigue, and depression by addressing social support in individuals with CAD. Individuals with severe fatigue should be identified early in their recovery so that further fatigue-relieving programs can be implemented. Some rehabilitation interventions such as psychological interventions may be effective tools in reducing fatigue in individuals with CAD following ACS who lack social support. Social support is especially important for maintaining compliance with rehabilitation program (Bramwell, 1990). Participation in social support programs should be promoted for these patients. During rehabilitation, there are opportunities for social activities.

Our results specifically showed that social support from friends was associated with most types of fatigue among individuals with CAD without depression symptoms while only social support from others was associated with most types of fatigue among individuals with CAD with depression symptoms. These findings are new and to the best of our knowledge there are no known studies disentangling association between subjective fatigue levels and social support in relation to depression symptomatology. We hypothesize that depressed and fatigued individuals with CAD may have a lack of energy due to fatigue as well as lost interest in their environment due to the depressive symptomatology to seek social support. While family and friends may still initiate their support for the depressed and fatigued individual due to the sense of care and mutual history, other people might not initiate the social contact first and may not recognize the need to support the fatigued and depressed individual with CAD.

The present study has several strengths, including large sample size, and the use of validated instruments. Despite of our consistent findings, several limitations should be discussed. The major limitation of our report is a cross-sectional design of the study and all information being completed in a single rehabilitation clinic. Thus, the results generated in a selective group of individuals. Particularly, most of the individuals were males, had NYHA-II functional class and all of them attended a single in-patient center, which may limit the generalization of our results (Narvaez Linares et al., 2021). Several other CAD risk factors were not addressed in this study, such as diabetes mellitus status, thyroid dysfunction, or possible sleep disorders, which might have influenced results. Future studies should explore the possible role of mediating factors that may affect the interplay between fatigue, depression symptoms, and perceived social support. However, in the statistical adjustment, we controlled for sex and NYHA functional class.

## Conclusion

The results of this study suggest that the fatigue and its features could be predicted by the perceived social support in individuals with CAD following ACS. While in individuals with CAD and with markedly expressed symptoms of depression higher levels of subjective fatigue are associated with mostly social support from others, higher levels of subjective fatigue in non-depressed individuals with CAD are significantly associated with mostly reduced social support from friends. Further studies may emphasize the effectiveness of evidence based psychological interventions during rehabilitation programs when high fatigue and low perceived support depending on the levels of depressive mood are being addressed.

## Data Availability Statement

The study dataset is available upon request to the authors and with the confirmation of Neuroscience Institute, Lithuanian University of Health Sciences.

## Ethics Statement

The studies involving human participants were reviewed and approved by the Biomedical Research Ethics Committee for Biomedical Research at Lithuanian University of Health Sciences. The patients/participants provided their written informed consent to participate in this study.

## Author Contributions

JB designed the study. NK, AB, and JG-S analyzed the data and drafted and edited the manuscript. All authors contributed to the article and approved the submitted version.

## Funding

This project has received funding from European Social Fund (project No. 09.3.3-LMT-K-712-19-0127) under grant agreement with the Research Council of Lithuania (LMTLT).

## Conflict of Interest

JG-S has served as a consultant at FACITtrans. In the past several years, JB has worked as a consultant to Cogstate, Ltd.

The remaining authors declare that the research was conducted in the absence of any commercial or financial relationships that could be construed as a potential conflict of interest.

## Publisher’s Note

All claims expressed in this article are solely those of the authors and do not necessarily represent those of their affiliated organizations, or those of the publisher, the editors and the reviewers. Any product that may be evaluated in this article, or claim that may be made by its manufacturer, is not guaranteed or endorsed by the publisher.
